# Quantitative assessment of paravalvular regurgitation following transcatheter aortic valve replacement

**DOI:** 10.1186/s12968-015-0134-0

**Published:** 2015-05-08

**Authors:** Gareth Crouch, Phillip J Tully, Jayme Bennetts, Ajay Sinhal, Craig Bradbrook, Amy L Penhall, Carmine G De Pasquale, Robert A Baker, Joseph B Selvanayagam

**Affiliations:** South Australian Health and Medical Research Institute, Adelaide, Australia; Department of Cardiothoracic Surgery, Flinders Medical Centre, Adelaide, Australia; Department of Cardiology, Flinders Medical Centre, Flinders Drive, Bedford Park, Adelaide, SA 5042 Australia; Flinders University of South Australia, Adelaide, SA Australia

**Keywords:** TAVI, Transcatheter, Cardiovascular magnetic resonance, Aortic valve, Paravalvular aortic regurgitation

## Abstract

**Background:**

Paravalvular aortic regurgitation (PAR) following transcatheter aortic valve implantation (TAVI) is well acknowledged. Despite improvements, echocardiographic measurement of PAR largely remains qualitative. Cardiovascular magnetic resonance (CMR) directly quantifies AR with accuracy and reproducibility. We compared CMR and transthoracic echocardiography (TTE) analysis of pre-operative and post-operative aortic regurgitation in patients undergoing both TAVI and surgical aortic valve replacement (AVR).

**Methods:**

Eighty-seven patients with severe aortic stenosis undergoing TAVI (56 patients) or AVR were recruited. CMR (1.5 T) and transthoracic echocardiography (TTE) were carried out pre-operatively and a median of 6 days post-operatively. The CMR protocol included regurgitant aortic flows using through-plane phase-contrast velocity. None/trivial, mild, moderate and severe AR by CMR was defined as ≤8%, 9-20%, 21–39%, >40% regurgitant fractions respectively.

**Results:**

Pre- and post-operative left ventricular ejection fraction (LVEF) was similar. Post-procedure aortic regurgitant fraction using CMR was higher in the TAVI group (TAVI 16 ± 13% vs. AVR 4 ± 4%, p < 0.01). Comparing CMR to TTE, 27 of 56 (48%) TAVI patients had PAR which was at least one grade more severe on CMR than TTE (Z = −4.56, p <0.001). Sensitivity analysis confirmed the difference in PAR grade between TTE and CMR in the TAVI group (Z = −4.49, p < 0.001).

**Conclusion:**

When compared to CMR based quantitative analysis, TTE underestimated the degree of paravalvular aortic regurgitation. This underestimation may in part explain the findings of increased mortality associated with mild or greater AR by TTE in the PARTNER trial. Paravalvular aortic regurgitation post TAVI assessed as mild by TTE may in fact be more severe.

## Background

There now exists extensive registry and clinical trial data demonstrating an increased incidence of paravalvular aortic regurgitation (PAR) following transcatheter aortic valve implantation (TAVI), and consequently, increased mortality over short term follow-up [1-5]. This is particularly pronounced when compared to the gold standard surgical aortic valve replacement (AVR). Despite recent improvements in both hardware and software, transthoracic echocardiographic (TTE) measurement of paravalvular aortic regurgitation (PAR) largely remains qualitative. This is particularly evident in TAVI associated PAR where patient factors such as airways disease, habitus and prior cardiac surgery often limit acoustic windows. Additionally the regurgitant jets are often multiple and eccentric making traditional echocardiographic regurgitation assessment techniques invalid. Despite these profound limitations echocardiography is still utilised as the primary assessment tool for PAR post TAVI. This has prompted the development of improved and standardized techniques such as those from the Valve Academic Research Consortium (VARC), however these have not been validated [6].

By contrast to echocardiography, cardiovascular magnetic resonance (CMR) is able to directly quantify aortic regurgitation with high accuracy and reproducibility by using the technique of phase-contrast velocity mapping. CMR is not affected by the location, number or nature of regurgitant jets or thoracic structural patient factors, and therefore offers an ideal technique for assessing the severity of TAVI associated PAR. CMR has previously been well validated in the quantitative assessment of aortic valve regurgitation [7-10]. The assessment of regurgitation severity is identical whether intravalvular or paravalvular. Furthermore the use of CMR in the early post-operative after TAVI or AVR to assess both valve and ventricular function has been validated [11].

To date there has been several studies utilizing CMR flow imaging to assess PAR in TAVI cohorts. The majority of these trials have compared CMR assessment to that of either qualitative or semi-quantitative echocardiography [12-14]. The limitations of these studies include small numbers, mixed valve types, and discrepant findings. Thus this we have attempted to address these limitations in our study design to further validate the use of CMR in PAR assessment and clarify the incidence of PAR in a single prosthesis type.

In a single-center prospective control trial, we assessed the extent of PAR in patients undergoing TAVI and high-risk AVR using this highly accurate and reproducible CMR technique. We compared the CMR PAR assessment with semi-quantitative TTE assessment, still the most used technique worldwide. We hypothesized that TTE would systematically underestimate the severity of PAR in TAVI patients compared with CMR. Furthermore this underestimation would be more pronounced in the TAVI group than in the AVR group owing to the complexity of the paravalvular regurgitation.

## Methods

### Ethics

This study was approved by the Human Research Ethics Committee of Flinders Medical Centre (Approval No. 237.11, 13 July 2011) and conducted in accordance with the Declaration of Helsinki. All patients gave written informed consent.

### Patient selection

Ninety patients with severe aortic stenosis undergoing either TAVI or high risk (STS Score >4, euroSCORE > 10) AVR were enrolled between June 2011 and July 2014. The decision to proceed with either procedural technique was made by the heart team at our institution based on clinical assessment. Three patients were excluded due to inadequate image quality (2 TAVI, 1 AVR), leaving 56 TAVI patients and 31 AVR patients (87 total).

### Procedure technique

All transcatheter valves were Edwards Sapien XT prostheses (Edwards Lifesciences, California USA) inserted via the femoral route. The AVR group all received bioprosthetic valves, access being via a median sternotomy using cardio-pulmonary bypass. Myocardial preservation and implantation techniques were similar in the surgical group. Four different tissue valve prostheses were used: Medtronic Mosaic® - Medtronic Inc, Minnesota USA; St Jude Medical Epic™ and Trifecta™, St Jude Medical Inc, Minnesota; Edwards Perimount Magna, Edwards Lifesciences, California USA.

### Imaging techniques

CMR (1.5 T Siemens Aera, Siemens - Germany) and transthoracic echocardiography (TTE, General Electric Vivid E9, GE Healthcare - UK) were carried out pre-operatively and post-operatively at a median of 5 and 6 days for TAVI and AVR respectively. Both CMR and echo were performed on the same day (consecutively), in random order and analysed by separate blinded operators.

**Figure 1:**
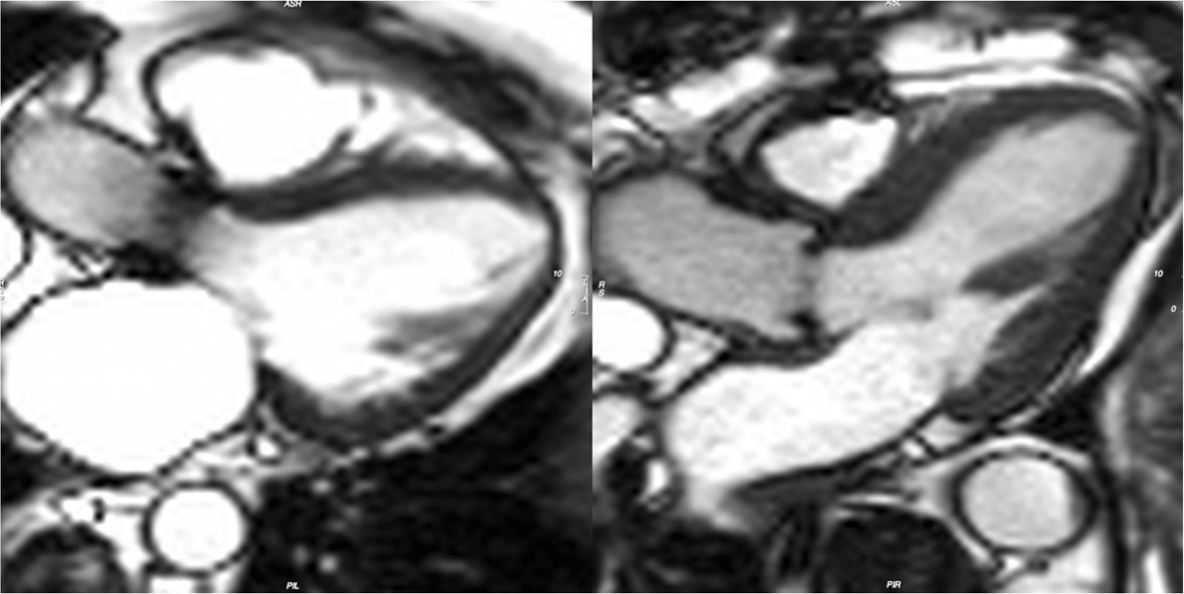
CMR Images **(a)** LVOT image post TAVI and **(b)** LVOT Image post AVR.

CMR images were analysed offline using commercially available software (CVI42, Circle CVI, Alberta, Canada). The CMR protocol consisted of standard LV short and long axis views (steady state free precession images) and forward and regurgitant aortic flows using through- plane phase-contrast velocity mapping (free breathing, retrospective gating) (Figure 1). The image plane was placed 0.5 cm above the aortic valve at end-diastole, but a position in the aortic root was maintained throughout the cardiac cycle. The severity of regurgitation by CMR regurgitant fraction was stratified according to published criteria: none/trivial <8%, mild 9 - 20%, moderate 21–39%, and severe >40% [13] [15]. The inclusion of a none/trivial grading (RF <8%) permits separation of those patients with clearly no or trivial regurgitation from those with mild regurgitation, as mild PAR has been demonstrated in the PARTNER cohort to be clinically significant. Conversion of CMR regurgitant fraction to a severity grading allowed direct comparison of imaging techniques.

Echocardiography was performed in the left decubitus position using commercially available Vivid E9 ultrasound machines (General Electric-Vingmed Ultrasound, Milwaukee, WI, USA). Data were analysed offline using EchoPAC PC Version 7 (General Electric-Vingmed Ultrasound) by 2 experienced echocardiography trained cardiologists. Aortic valve regurgitation was graded using a combined approach of semi-quantitative and qualitative parameters. For post-operative assessment this included visual assessment of the number of jets, jet width and the circumferential extent for paravalvular regurgitation, as per existing guidelines and the more recent VARC-2 criteria [6,16]. Regurgitation was classified as none/trivial ‘0’, mild ‘1’, moderate ‘2’ and severe ‘3’ [6]. Parasternal short and long-axis views and five chamber views were used to assess the quantity and qualities of AR jets as well as the extent into the ventricle. Jet width was measured just below the ventricular side of the valve stent frame for PAR sufficient to avoid artifact and graded according to % width of the LVOT. The circumferential extent (%) of PAR was assessed in the parasternal short-axis view and graded according to the following definition: none/trivial (no or pinpoint jet), mild (jet <10%), moderate (10%– 29%) and severe (≥30%) [6]. Aortic flow reversal was assessed from multiple windows including suprasternal notch and sub-costal views, and used for both PAR and pre-operative AR assessment. Pre-operative AR was assessed using standard imaging techniques [17]. Where disagreement existed between echocardiographic parameters an additional blinded assessor was utilised and a consensus reached.

### Statistical analysis

Data analysis was performed with SPSS® 20.0 (SPSS Inc., Chicago, IL). Descriptive comparisons between TAVI and AVR included the independent samples *t*-test and the chi-square statistic with Fisher’s exact test as appropriate. Agreement between grade of PAR ascertained by CMR and TTE was estimated with the Wilcoxon signed-rank test and Bland-Altman analysis. These were performed separately for TAVI and AVR in sensitivity analysis. We also applied the Kappa statistics to determine the agreement on grading of AR severity consistent with Altiok et al. All statistical tests were two-tailed, an alpha value *p* < .05 was considered statistically significant and no adjustment was made for multiple comparisons.

## Results

A total of 87 patients were recruited, 56 TAVI and 31AVR. Although TAVI patients were older, STS scores were similar between the groups (Table 1). Other comorbidities were similar except reoperation was more common in the TAVI group. Early post-operative CMR and TTE were conducted at a median of 5 days for TAVI and 6 days for AVR. Mean preoperative left ventricular (LV) and right ventricular (RV) ejection fractions (EF) were similar in the 2 groups using CMR (AVR 68% ± 16 vs. TAVI 65% ± 16, p = NS). Post-operative LVEF was also similar in both groups (AVR 66% ± 16 vs. TAVI 66 ± 16, p = NS).

**Table 1:** **Patient characteristics**

	**AVR**		**TAVI**		***P***
	**n = 31**		**n = 56**		
Age (SD)	80	4	84	6	<.01
Male (%)	15	48	34	61	.27
STS Score (SD)	8.7	7.7	8.9	5.4	.45
Hypertension (%)	26	84	50	90	.47
Previous MI (%)	5	16	12	21	.55
COPD (%)	15	48	23	41	.51
Renal Impairment (%)	11	35	18	32	.76
Atrial Fibrillation (%)	8	26	20	36	.34
Diabetes (%)	15	48	22	39	.42
Redo (%)	0	0	23	41	<.01
Previous CVA/TIA (%)	6	19	17	31	.27
NYHA Class (SD)	2.7	0.6	2.7	0.7	.42
PAH (%)	11	36	14	25	.30

BMI – Body Mass Index; CAD – Coronary Artery Disease; COPD - Chronic Obstructive Pulmonary Disease; MI – Myocardial Infarction; Neuro CVA- Previous TIA or CVA; PAH – Pulmonary Artery Hypertension; PCI - Percutaneous coronary intervention; Redo – Previous Cardiac Surgery; STS – Society of Thoracic Surgeons Score.

Pre-procedure aortic valve regurgitation was similar between AVR and TAVI when compared using CMR regurgitant fraction (AVR 14% ± 15 vs. TAVI 18 ± 16, p = 0.14). Comparing pre-procedure aortic valve regurgitation grades between imaging techniques, CMR and TTE demonstrated near identical mean values for TAVI (CMR1.1 ± 1.0 vs. TTE 1.0 ± 0.8, p = 0.21) and AVR groups (0.8 ± 0.9 vs. 0.7 ± 0.7, p = 0.36). All regurgitation was valvular in nature and less severe than the predominating stenosis. The Wilcoxon signed-rank test did not indicate a significant difference between CMR AR grade and TTE (Z = −.323, p = .73).

All post-procedure regurgitation was paravalvular in nature when assessed by both TTE and intra-operative transoesophegeal echocardiography (TOE). Post-operative mean regurgitant fraction using CMR was higher in the TAVI group when compared to the AVR group (TAVI 16% ± 13 vs. AVR 4% ± 4 p < 0.001). In the AVR group only four patients had PAR, which was mild in the majority of cases (Table 2).

**Table 2:** **Comparison of post-operative PAR severity**

***AVR***	***None/Triv.***	***Mild***	***Moderate***	***Severe***
**CMR**	27 (87%)	3 (10%)	1 (3%)	0
**Semi-Quant. TTE**	28 (90%)	3 (10%)	0	0
***TAVI***				
**CMR**	15 (26%)	22 (39%)	17 (30%)	3 (5%)
**Semi-Quant. TTE**	21 (38%)	32 (57%)	1 (2%)	0 (0)%)

Between groups Z = −4.56, p < .001.

Kappa = .41 SE = .07.

**Figure 2:**
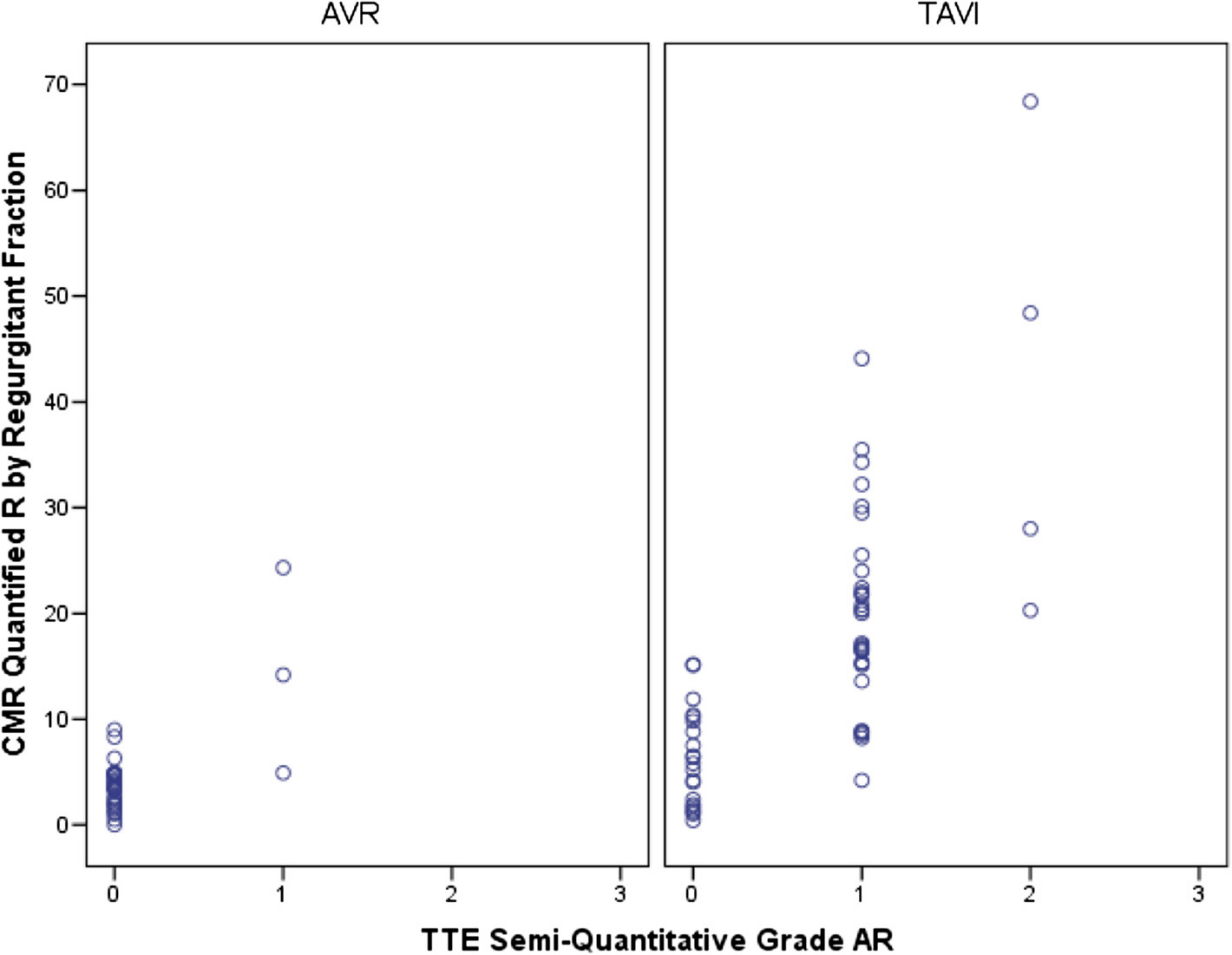
Correlation CMR regurgitant fraction and semi-quantitative TTE for AVR and TAVI.

In the TAVI group 52% of patients (29/56) had semi-quantitative TTE findings which graded PAR at a different value to CMR. In 93% (27/29) of these cases the PAR was graded at a lesser value by TTE (Z = −4.56, p < .001). Sensitivity analysis confirmed that the difference in PAR grade was evident in the TAVI group (Z = −4.49, p < .001) but not the AVR group (Z = −1.00, p = .32). The CMR grade (continuous) is plotted by TTE grade in Figure 2.

## Discussion

Our principle findings show that: (1) using CMR quantitative analysis there was significantly more PAR in the TAVI group than AVR group; (2) semi-quantitative transthoracic echocardiography underestimates the degree of paravalvular AR when compared to quantitative CMR; (3) there was no difference between CMR and TTE in pre-procedure native valvular aortic regurgitation. To the best of our knowledge this is the first time CMR assessment of TAVI associated PAR has been reported with a matched surgical AVR control group. Additionally this is the largest series describing PAR evaluation using CMR of a single transcatheter valve type (Edwards Sapien XT), which holds relevance when transcatheter programs are selecting their preferred devices.

CMR has been specifically compared to echocardiography in the assessment of TAVI associated PAR in three prior studies. Ribeiro et al. compared CMR and TTE (VARC-2 criteria) in 42 patients who had Edwards Sapien XT transcatheter valves, finding TTE underestimated PAR in 62% of cases [14]. Conversely Altiok and colleagues compared CMR to 2D and 3D TTE (VARC-2 criteria) in 71 patients finding disagreement between VARC-2 TTE PAR assessment and CMR in only 18% of patients studied [12]. A smaller study of 16 patients with the CoreValve prosthesis [18] showed CMR correlated well with invasive catheter based assessment and poorly with TTE, which again appeared to underestimate the regurgitation [18]. Further to the assessment studies of PAR severity, there is now published data on the potential clinical consequences the underestimation by TTE may have. In a small series Hartlage et al. conducted CMR evaluation in 23 patients with heart failure symptoms and PAR post TAVI, finding CMR reclassified the PAR in nearly 50% of cases [13]. Notwithstanding the lower incidence of PAR severity in one of these studies the findings generally concur with those of our prospective control group study, that even semi-quantitative TTE assessment significantly underestimates the degree of PAR. However, our study differs from these prior studies in also demonstrating the significantly greater incidence of PAR in TAVI patients compared with a similarly matched group of AVR patients using quantitative CMR assessment.

The potential importance of CMR quantification of PAR is underscored by the finding that, after converting CMR regurgitant fraction to regurgitant grade, 74% of patients were classified as having mild or greater PAR. This compares to a range of 21 – 61% reported in prior echocardiographic based studies [19]. Furthermore, our finding that 35% of TAVI patients had moderate or severe PAR, exceeds the echocardiographic incidence of moderate or greater PAR in the PARTNER trial reported at 12% [20] and other trials reported at 3.8 to 13% [21,22]. This potential systemic underestimation of postoperative PAR by TTE was supported by several other key findings. Firstly, 48% of TAVI patients had a regurgitant grade lower on semi-quantitative TTE than CMR. Secondly, the discordance between CMR and TTE was evident by significant Wilcoxon rank-sum test, and finally sensitivity analysis corroborated that the difference in TTE and CMR was constrained only to the TAVI group.

The finding of significant corroboration between CMR and TTE in the assessment of pre-procedure (native valve) aortic regurgitation offers two key insights. The first is that it validates CMR techniques for assessing aortic valve regurgitation with those of TTE. Secondly it objectively confirms that the echocardiographic techniques which are readily applicable to native valve or intravalvular regurgitation are inherently unreliable in the transcatheter PAR setting. Our findings regarding pre-procedure regurgitation are supported by earlier work, which also found consistent agreement between TTE and CMR in pre-procedure aortic valve regurgitation [18]. Despite the paucity of data in assessing PAR, CMR has been well validated in the assessment of aortic valvular regurgitation [23]. It is recognized as being highly reproducible, less susceptible to artifact, quantitative and offering concurrent gold standard ventricular assessment.

Paravalvular aortic regurgitation (PAR) remains a significant and underestimated issue for transcatheter valves despite the impending arrival of third generation devices. Until TAVI can match surgical PAR rates and hence reduce associated increased mortality AVR will remain first line therapy. Furthermore, there is now a large population of patients with transcatheter valves who are affected by mild or greater PAR. It is widely accepted patients with PAR face increased morbidity and mortality and there may be an opportunity to intervene either medically or procedurally should the accurate assessment offered by CMR be broadly adopted [24]. The association of *mild* PAR with increased mortality was a particularly unexpected finding of the PARTNER trial [24]. We speculate from our findings that whilst the causality component of this finding is correct the assessment of mild PAR is not. We suggest that in fact the mild PAR is in fact moderate or greater in severity and this occurs as a result of underestimation by transthoracic echocardiography.

## Conclusion

We propose that PAR can be easily, reproducibly and accurately assessed using CMR. The increased incidence of PAR with TAVI compared to AVR is confirmed using quantitative CMR assessment. Furthermore we offer a hypothesis that the association of mortality with mild AR in the PARTNER trial may be due to limitations of echocardiography causing underestimation of the degree of PAR.

## Abbreviations

PAR: Paravalvular Aortic Regurgitation
AS: Aortic Stenosis
AVR: Aortic Valve Replacement
CMR: Cardiovascular Magnetic Resonance
LV: Left Ventricular
LVEDV: Left Ventricular End-Diastolic Volume
LVESV: Left Ventricular End Systolic Volume
RV: Right Ventricular
TAVI: Transcatheter Aortic Valve Replacement
TOE: Transoesophegeal Echocardiography
TTE: Transthoracic Echocardiography
